# Hikikomori Risk in the UK

**DOI:** 10.1177/00207640251348058

**Published:** 2025-07-05

**Authors:** Gregory Gorman, Alison Bacon, Jon May, Stephen Minton

**Affiliations:** School of Psychology, University of Plymouth, UK

**Keywords:** Hikikomori, social withdrawal, HRI-24, HRI short, modern type depression

## Abstract

**Background:**

Hikikomori syndrome involves voluntary withdrawal from social life, school and work, with onset typically in young adulthood. Hikikomori risk has not been examined in the UK, and these studies aimed to validate and refine the Hikikomori Risk Inventory-24 (HRI-24) screening tool in UK young adults.

**Methods/Results:**

In Study 1, participants (*n* = 341) completed the HRI-24. Item analysis and confirmatory factor analysis resulted in a shorter 13-item HRI (HRI-13), which demonstrated a perfect correlation with the full HRI-24. Both the HRI-13 and HRI-24 showed strong convergent and divergent validity, correlating with depression, anxiety, avoidant coping and negative early life factors.

Study 2 (*n* = 228) found a significant positive correlation between HRI-13 scores and modern-type depression, typified by social avoidance and often comorbid with technology-based addictions.

**Conclusion:**

Both HRI-13 and HRI-24 effectively capture hikikomori risks related to negative affect and anxiety but may have limitations in identifying risks unrelated to negative affect.

## Introduction

Hikikomori, a Japanese term meaning to ‘pull back’ and ‘seclude oneself’, refers to individuals who deliberately withdraw from social life, including school and work, often staying in their rooms or homes (Tan et al., 2021). This psychosocial condition, increasingly recognized in recent years, typically affects adolescents and young adults. While some studies report an average age of onset between 20 and 27 years (Pozza et al., 2019; Teo et al., 2015), others suggest it begins earlier, around age 13 (Malagón et al., 2014). Such individuals manage stress and cope with negative emotions or external demands through maladaptive and prolonged withdrawal.

Criteria for hikikomori syndrome include: (i) staying home most of the day; (ii) avoiding social interactions (e.g. school, work); and (iii) prolonged isolation for at least 6 months. Kato et al. (2020) added that the isolation must cause significant distress or impairment. Originally considered a culture-bound syndrome, hikikomori has been mainly studied in Japan, where it first emerged, but has also been documented in countries like Oman, Spain, Korea, the US, Italy, India and France (Benarous et al., 2022; Bowker et al., 2019; Kato et al., 2012; Ranieri, 2015; Teo et al., 2015).

Depression and shame often co-occur with hikikomori (Yong & Nomura, 2019). A decline in social status or self-perceived unattractiveness can trigger shame, which tends to be self-directed, leading to avoidance strategies (Gilbert, 1997). Other risk factors include early life experiences and resources. Varnum and Kwon (2016) speculated that the spread of hikikomori could stem from unpredictable childhoods or early trauma in resource-rich environments with generous social policies. Peer rejection and childhood adversity have also been linked to hikikomori (Frankova, 2019; Hattori, 2006; Stip et al., 2016). Additionally, social withdrawal is more common in individuals from middle-class families or those with good socioeconomic status (Coeli et al., 2023; Wong et al., 2019).

Finally, it is worth noting that while some studies have reported gender differences in the prevalence of hikikomori (Chan, 2023; Kato et al., 2019; Nonaka & Sakai, 2023; Pozza et al., 2019). A review by Nonaka et al. (2022), with a total sample size of 4,744 participants, found that 76.49% were male, indicating a strong male predominance in hikikomori populations. A subsequent review in 2025 by Nonaka et al. similarly observed a higher proportion of men than women, with sex ratios differing between Japan and other countries, suggesting potential cultural influences. Nonaka et al. (2025) also noted the overlap with other psychiatric conditions, particularly autism spectrum disorder (ASD) and social anxiety, which may contribute to gender differences in reported cases – given that ASD is more commonly diagnosed in men, while social anxiety is more prevalent in women.

### Subtypes and Measurement of Hikikimori

Despite over two decades of research, there is no consensus on whether hikikomori is a distinct syndrome or a comorbid condition. Amendola’s (2024) review highlighted significant variations in definitions and interpretations, complicating its classification. Many definitions lack clear criteria for functional impairment, making it difficult to distinguish hikikomori from other conditions like social anxiety, depression or adjustment disorder. There is ongoing debate on whether hikikomori is a syndrome or a transdiagnostic behaviour, influenced by underlying mental health issues (Li & Wong, 2015; Teo et al., 2015; Teo & Gaw, 2010).

Kato et al. (2019) identified psychiatric disorders most commonly co-occurring with hikikomori, such as psychosis, social anxiety, depression, PTSD and avoidant personality disorder. Despite symptom overlap, these conditions are not identical to hikikomori (Kondo et al., 2008), and subtypes have been proposed. Malagón-Amor et al. (2018) identified two subgroups: an anxiety-affective subgroup (characterized by social anxiety and depression) and another with psychotic, drug use and personality disorders. Additional distinctions include individuals who desire a hikikomori lifestyle (egosyntonic) versus those who feel distressed by it (Tolomei et al., 2023).

The anxiety-affective subtype is relevant to the Hikikomori Risk Inventory (HRI-24; Loscalzo et al., 2022), a measure that focuses on negative affect. However, it may not capture the psychiatric subtype of hikikomori, focusing mainly on affective and anxious symptoms. Another limitation is the HRI-24’s English phrasing, as it was initially developed in Italian and validated in a back-translated Japanese version, but not in an English-speaking sample. This is the first study to administer and validate the HRI-24 in a UK population, or to conduct item quality analysis. Additionally, despite the focus on negative affect, associations between HRI-24 scores and those on standardised clinical measure of depression and anxiety, or measures of neuroticism, have yet to be examined. The present study will also examine the association with measures of coping behaviours, early-life experiences, status attitudes and feelings of shame.

When examining the role of coping behaviours – it is conceptualised as the cognitive and behavioural strategies individuals use to manage stress and regulate affect (Finset et al., 2002) – in relation to hikikomori risk. Coping is particularly relevant to this construct because individuals vulnerable to social withdrawal may rely more heavily on avoidant coping styles, which can perpetuate isolation and affective dysregulation (Ottenbreit & Dobson, 2004). Avoidant coping has been associated with negative affect, neuroticism and sensitivity to social threat – factors commonly implicated in hikikomori presentations (Costa & McCrae, 2006; Gilbert, 1997). By exploring the associations between HRI scores and both maladaptive (e.g. avoidance) and adaptive (e.g. approach) coping strategies, we aim to clarify how these mechanisms may reflect the psychological profile of individuals at risk of hikikomori, and provide validity for the HRI-24 measure. We predict a positive relationship between hikikomori risk and measures of negative affect, neuroticism, shame and early-life unpredictability. However, a negative relationship with status ambition is expected. Higher early-life resources, suggested as a risk factor in Japan, will also be tested in the UK context. A second study will refine the HRI-24 and further validate it by examining its relationship with contemporary depression types.

## Study 1

### Methodology

#### Participants

A total of 341 participants aged 18–25 were recruited, reflecting a convenience sample of young adults. 141 were recruited from an established student research participant pool at the authors’ university and received partial course credit for their time. An additional 200 UK nationals aged 18-25 were recruited from Prolific (an online survey platform), and these participants were each paid £1.50. 181 participants were men and 160 were women. Age data were not collected at the individual level, as participants were screened to fall within the 18–25 age range during recruitment. Therefore, mean and standard deviation values for age are not available. Of these, 55.7% were in education (*n* = 190), 31.7% were employed (*n* = 108), 4.1% were self-employed (*n* = 14) and the remaining 8.5% were unemployed (*n* = 29).

The sample of 341 participants is sufficient for psychometric analyses, including confirmatory factor analysis (CFA) and item response theory (IRT). MacCallum et al. (1999) recommend at least 200 participants for CFA, and Jiang et al. (2016) suggest that 200 is also adequate for IRT, though larger samples improve parameter precision. Dai et al. (2021) ran a simulation study and found that at least 300 participants are needed for stable item parameter estimation, particularly for polytomous IRT models like the graded response model (GRM).

### Procedures and Measures

A survey was distributed online via Qualtrics. Participants completed the following measures, with Cronbach’s alpha for the current sample indicated.

### Hikikomori Risk Inventory (HRI-24)

The HRI-24 assesses the risk of hikikomori in both Eastern and Western countries (Loscalzo et al., 2022). It consists of 24 items across five subscales: Anthrophobia (α = .88), Agoraphobia (α = .88), Paranoia (α = .79), Lethargy (α = .84) and Depression (α = .89), with an overall α = .94. The scale has been validated in Italy and Japan, showing a robust factor structure (CFI = 0.94, RMSEA = 0.057). The HRI-24 is scored on a 5-point Likert scale (1 = Strongly disagree to 5 = Strongly agree) to create a total score by summing all items.

### Patient Health Questionnaire (PHQ-9)

The PHQ-9 screens for depression, based on DSM-IV criteria (Spitzer et al., 1999). It demonstrated excellent internal consistency in this study (α = .89).

### General Anxiety Disorder (GAD-7)

The GAD-7 assesses General Anxiety Disorder. It showed excellent internal consistency (α = .92) in this study (Spitzer et al., 2006).

### Brief Approach/Avoidance Coping Questionnaire (BACQ)

The BACQ assesses coping behaviours through two main subscales: approach coping (α = .73) and avoidance coping, which further divides into diversion (α = .55) and withdrawal (α = .71; Finset et al., 2002). The questionnaire consists of 12 items: six for approach coping, three for diversion, and three for withdrawal. Both diversion and withdrawal are distinct subscales within the avoidance coping category.

### Neuroticism Scale (NEO Personality Inventory – UK)

The neuroticism scale measures traits related to negative affect (Costa & McCrae, 2006). It includes six facets: anxiety (α = .72), anger/hostility (α = .77), depression (α = .82), self-consciousness (α = .72), impulsiveness (α = .56) and vulnerability (α = .81). There are eight items per subscale, with a total of 48 items in the neuroticism scale.

### Early Life Unpredictability and Socioeconomic Status

Unpredictability was assessed with three items (α = .80) about childhood chaos (Griskevicius et al., 2011). Childhood socioeconomic status was measured with three items (α = .83) on resource availability during childhood (Mittal et al., 2015).

### Status Anxiety and Ambition

Status anxiety (α = .90) measures concerns about status decline (e.g. fear of being stuck in one’s position), while status ambition (α = .87) assesses the desire to improve social standing (Keshabyan & Day, 2020). Both are measured using five items.

### External and Internal Shame Scale (EISS)

The EISS measures internal and external shame. It consists of eight items, with Cronbach’s alpha of .90 for total shame, .82 for external shame and .82 for internal shame (Ferreira et al., 2022).

#### Analysis

This study employs Item Response Theory (IRT) alongside Confirmatory Factor Analysis (CFA) to evaluate the validity of the HRI-24, item performance, and develop a refined version of the HRI. The analysis combines Mokken analysis, IRT modelling and content analysis, with each method complementing the others. The IRT approach provides statistical insights into item performance, while the content analysis assesses item validity. The first two steps of the item quality analysis were conducted using the Mokken (Sijtsma & van der Ark, 2017) and MIRT packages in R (Chalmers, 2012).

Mokken analysis estimates the scalability for each item and the overall scale, with the scalability coefficient (H) indicating how each item contributes to the scale. Items with an H score of 0.0 represent no correspondence, while an H = 1 indicates perfect correspondence. Recommended cut-offs for items are ⩽0.3 (weak), 0.3 ⩽ H < 0.4 (medium) and ⩾0.5 (strong) (Sijtsma & Molenaar, 2002).

A two-parameter graded response model (2PL GRM) was applied to estimate item parameters, comparing person measures (likelihood to endorse) with item location (difficulty). Parameter estimation was conducted at the full 24-item scale, ensuring a stable foundation for model estimation. Item Information Functions (IIFs) were then examined at the subscale level to assess how well individual items discriminated between individuals at different levels of Hikikomori risk within each domain. Given that subscales contained fewer than five items (with the subscales of Anthropophobia, Agoraphobia and Lethargy having only four items), IIF estimates may be less stable, and results should be interpreted as exploratory (Dai et al., 2021). These findings were considered alongside other psychometric analyses to inform decisions regarding potential refinements to the HRI-24. Analyzing the subscales separately helps identify specific items that perform optimally or suboptimally across different dimensions of Hikikomori risk, providing a more nuanced understanding of the scale’s psychometric properties.

##### Unidimensionality Testing

To justify the application of Item Response Theory (IRT), we tested the assumption of unidimensionality using multiple methods. First, we conducted an Exploratory Factor Analysis (EFA) and parallel analysis to explore the underlying factor structure of the scale. Following this, we evaluated whether a unidimensional model would provide an adequate fit to the data.

Additionally, we performed a bifactor analysis, which separates each item’s variance into a general factor and specific subscale factors (Reise, 2012; Rodriguez et al., 2016). This approach allowed us to assess whether a single, overarching hikikomori risk dimension could meaningfully explain the responses, despite the presence of subscale-specific factors. The bifactor model provides several key indicators for evaluation, including eigenvalues, which reflect the amount of variance explained by each factor and help identify the dominance of a general factor (Reise, 2012; Rodriguez et al., 2016). We also computed the Omega Hierarchical coefficient, which indicates the proportion of reliable variance in the total scores attributed to the general factor (McDonald, 1999; Zinbarg et al., 2005). Furthermore, we examined Explained Common Variance (ECV), which quantifies the proportion of variance in the items explained by the general factor, excluding subscale-specific variance (Rodriguez et al., 2016). Finally, we assessed model fit indices for both the unidimensional and bifactor models to compare their adequacy in explaining the data. These indicators collectively help determine whether the general factor adequately explains the variance across items and supports the use of a unidimensional Item Response Theory (IRT) model (Reise, 2012; Reise et al., 2010).

##### Item Content Analysis

An item content analysis was conducted to examine the face validity of the questionnaire, ensuring each question encapsulated distinct aspects of the intended construct.

##### Confirmatory Factor Analyses and Correlations

The HRI-24 dataset underwent Confirmatory Factor Analysis (CFA) to evaluate both the unifactorial model and sub-scale structures for a UK population. Model fit was assessed using degrees of freedom, Scaled Chi-Square, CFI, TLI and SRMR, with acceptable thresholds: CFI/TLI ⩾ 0.90/0.95, RMSEA ⩽ 0.08/0.06 and SRMR ⩽ 0.08/0.06 (Bentler, 1990; Browne & Cudeck, 1992; Hu & Bentler, 1999; Steiger, 1990). CFA was performed with the lavaan package (Rosseel, 2012) and semTools (Jorgensen et al., 2018).

To evaluate the convergent and divergent validity of the Hikikomori Risk Inventory (HRI-24) and its shortened version (HRI-Short), we examined correlations with a range of theoretically relevant constructs in a UK sample. We hypothesised positive correlations with depression (Spitzer et al., 1999), generalized anxiety (Spitzer et al., 2006), neuroticism (Costa & McCrae, 2006), avoidance coping (Finset et al., 2002), early life unpredictability (Mittal et al., 2015), status anxiety (Keshabyan & Day, 2020), and shame (Ferreira et al., 2022; Gilbert, 1997), as these constructs have been associated with withdrawal, affective dysregulation and sensitivity to social threat (Gilbert, 1997; Price et al., 1994; Wilson & Daly, 1985). In particular, research has linked status anxiety and shame to feelings of defeat, loss of social attractiveness, and withdrawal – processes relevant to hikikomori presentations (Vandello & Bosson, 2013). Neuroticism and avoidance coping are similarly implicated in affective disorders and maladaptive withdrawal strategies (Ottenbreit & Dobson, 2004). We expected negative correlations with approach coping and status ambition, as these traits reflect proactive, goal-directed behaviour and improved social functioning (Tiet et al., 2006), which may buffer against social withdrawal.

## Results

### Item Analysis and Unidimensionality Tests

When testing the assumption of unidimensionality for the application of Item Response Theory (IRT), the unidimensional model did not achieve satisfactory fit (see Table 1). Exploratory Factor Analysis and parallel analysis suggested a five-factor solution, aligning with the original scale design (Fabrigar et al., 1999; Horn, 1965). Despite this, a unifactorial approach may still be defensible based on the strength of a general underlying construct – supported by bifactor analysis.

**Table 1.** Goodness of fit tests.

Fit Index	Unifactorial	HRI scales	HRI.IRT
*df*	252	242	56
chisq.scaled	1,390.01	437.42	74.37
cfi.robust	.72	.95	.99
tli.robust	.69	.95	.99
rmsea.robust	.12	.05	.03
srmr	.09	.05	.03

*Note. df* = degrees of freedom; CFI = Comparative Fit Index; TLI = Tucker-Lewis Index; SRMR = standardized root mean square residual; RMSEA = root mean square error of approximation.

Bifactor analysis revealed a dominant general factor, with an eigenvalue of 9.91 – substantially larger than the eigenvalues of subsequent factors – indicating a meaningful general dimension (Reise, 2012; Rodriguez et al., 2016). The Omega Hierarchical coefficient was 0.77, suggesting that 77% of the reliable variance in total scores could be attributed to the general factor (McDonald, 1999; Zinbarg et al., 2005). The Explained Common Variance (ECV) was 0.56, further supporting the presence of a general dimension across items (Rodriguez et al., 2016).

Model fit indices for the bifactor model were excellent (RMSEA = 0.038; 90% CI = 0.028–0.047) and significantly better than the unidimensional model (RMSEA = 0.123). While the multidimensional structure provides important insights at the subscale level, these findings suggest that the scale is sufficiently dominated by a general factor to justify the use of a unidimensional IRT model – particularly for applications such as screening, total score interpretation or short-form development (Reise, 2012; Reise et al., 2010).

### Mokken Analysis

The Mokken scalability analysis demonstrated that most items behaved well within the HRI-24, but the overall scalability H were below the recommended 0.5 for a strong scale. However, several items placed below the recommended cut-off of 0.3 (see Table 2). All *Anthropophobia*, *Agoraphobia*, *Lethargy* and *Depression* items performed moderately, placing between 0.4 and 0.5 H, except for LE 4. Many of the *Paranoia* items were weak, some scoring below the cut off of 0.3.

**Table 2.** Scalability for each HRI-24 item (confidence intervals of 95%).

Item	*M*	*SD*	Scalability	CIs (0.95)
*Anthropophobia*				
1. I feel severely anxious when I meet people outside	2.86	1.14	0.47	[0.41, 0.52]
2. I am uncomfortable when I am in touch with others	2.62	1.12	0.44	[0.38, 0.49]
3. I have great difficulty in participating in social activities	2.82	1.15	0.46	[0.41, 0.52]
4. I am afraid of social relationships	2.55	1.13	0.47	[0.41, 0.52]
*Agoraphobia*				
1. I feel very anxious when I am in a closed space	2.35	1.18	0.46	[0.41, 0.52]
2. I am afraid of being in crowded places	2.77	1.26	0.43	[0.38, 0.49]
3. I avoid going to public places when I go out alone	2.49	1.20	0.44	[0.39, 0.50]
4. When I am in crowded places, I feel some strange sensations.	2.57	1.25	0.44	[0.39, 0.50]
*Paranoia*				
1. I am diffident and suspicious of others	2.93	0.78	0.26	[0.17, 0.35]
2. Confiding with others is dangerous because people might then reveal my secrets	2.98	1.18	0.31	[0.24, 0.38]
3. I am afraid of being deceived by others	3.25	1.14	0.41	[0.35, 0.48]
4. I prefer doing things on my own because I do not trust people	2.92	1.12	0.36	[0.30, 0.43]
5. People are willing to do anything to achieve their goals	3.55	0.95	0.26	[0.18, 0.33]
6. I only trust myself	2.82	1.12	0.33	[0.26, 0.40]
7. If others do me a favour, I do not believe in their good intention	2.32	1.02	0.41	[0.35, 0.48]
*Lethargy*				
1. I feel tired and fatigued	3.71	1.08	0.44	[0.38, 0.50]
2. I manage to do my activities with difficulty because I feel powerless	2.55	1.12	0.47	[0.41, 0.52]
3. I often feel weak and lacking in energy	3.24	1.22	0.48	[0.42, 0.53]
4. I sleep many hours a day because I often feel weak	2.67	1.24	0.39	[0.33, 0.45]
*Depression*				
1. There are moments in which nothing seems important	3.32	1.23	0.48	[0.42, 0.54]
2. Nothing is able to really thrill me	2.51	1.17	0.44	[0.39, 0.50]
3. I rarely feel positive emotions	2.33	1.13	0.47	[0.42, 0.53]
4. I feel dispirited and hopeless for the future	2.61	1.22	0.49	[0.44, 0.54]
5. I feel a sense of inner emptiness	2.77	1.26	0.49	[0.44, 0.54]
Total	2.81	1.14	0.43	[0.38, 0.47]

### Graded Response Model

The 2PL GRM analysis (Table 3) showed significant variation in item discrimination, especially in the Paranoia subscale, with many items below 1. Most items had strong factor loadings and moderate correlations with withdrawal. *Anthropophobia* and *agoraphobia* items performed similarly, but *Anthropophobia* showed higher correlations with withdrawal. *Lethargy* items varied, with Items 2 and 3 being more informative. *Depression* items generally performed well, though Items 1 and 2 were less informative. Items with the highest discrimination and information also performed best in the Mokken scalability analysis, showing higher H coefficients.

**Table 3.** Item information and discrimination, factor loadings and correlation with BACQ withdrawal.

Items	Rel.info%	Discrimination	CFA load.lv	CFA.Std.all	Withdrawal (*r*)
Anthropophobia 1	5.36	1.77	.80	.70	.53
Anthropophobia 2	4.8	1.65	.73	.66	.51
Anthropophobia 3	5.29	1.77	.80	.70	.53
Anthropophobia 4	5.69	1.88	.78	.70	.55
Agoraphobia 1	4.53	1.67	.78	.67	.46
Agoraphobia 2	3.81	1.47	.80	.64	.41
Agoraphobia 3	4.23	1.59	.78	.65	.43
Agoraphobia 4	4.16	1.59	.82	.65	.45
Paranoia 1	0.74	0.57	.24	.3	.22
Paranoia 2	1.44	0.78	.49	.41	.28
Paranoia 3	2.87	1.16	.63	.55	.42
Paranoia 4	2.42	1.04	.56	.49	.34
Paranoia 5	0.86	0.60	.31	.33	.23
Paranoia 6	2.01	0.93	.51	.46	.33
Paranoia 7	3.58	1.38	.58	.57	.41
Lethargy 1	3.75	1.43	.67	.62	.5
Lethargy 2	6.31	2.05	.79	.71	.59
Lethargy 3	5.52	1.91	.85	.70	.56
Lethargy 4	3.48	1.37	.72	.58	.42
Depression 1	4.7	1.72	.82	.67	.58
Depression 2	4.71	1.68	.78	.67	.54
Depression 3	5.93	2.02	.78	.70	.59
Depression 4	7.06	2.32	.92	.78	.64
Depression 5	6.73	2.26	.95	.75	.61

*Note.* CFA loadings are from a unifactorial model, meaning this theoretically loadings should observe how well an item loads onto hikikomori risk.

### Item Information Function

The IIF analysis was done at a sub-scale level, to provide nuance that each item is conveying unique information to their respective domains (see Supplemental Figures S1–S5). The curvature of items overlaps for most sub-scales, which may indicate redundancy in some items, and most information, is obtained from person measures around the middle. *Anthropophobia*, *agoraphobia* and *depression* subscales showed decent curve areas, though *depression* items 1 and 2 were shallower. The Paranoia subscale had problematic items, particularly items 1 and 5. For *lethargy*, only Item 3 provided substantial information, while the others did not, indicating superior discrimination for Item 3.

### Content Analysis of Each Subscale

The contents of each item, along with the brief analysis can be seen in the supplementary material (Supplemental Table S1).

#### Anthropophobia

Captures fear of people and social unease. Question 2’s ‘in touch’ phrasing lacks clarity. The scale focuses primarily on fear of people although it does omit symptoms like negative self-comparison and low self-esteem/worth.

#### Agoraphobia

Covers fear of open spaces, physical symptoms and public but enclosed areas, encompassing anxiety, symptoms and avoidance. Questions 1 and 2 relate to a similar concept of being in public spaces.

#### Paranoia

Several issues arise from question phrasing. ‘Diffident’ in Question 1 is inaccessible, while Question 2 is a double-barrelled question, assuming secrecy and people wanting to uncover one’s secrets. Most questions address the ‘distrust’ aspect of paranoia. Notably absent are elements like feeling watched, interpreting events as threats and feeling persecuted.

#### Lethargy

Items revolve around tiredness, emphasizing a single aspect to the construct. Item 3, highlighting the frequency of tiredness with ‘often,’ seems best. Question 2 might relate more to self-efficacy than to lethargy, as it refers to feelings of powerlessness.

#### Depression

Items effectively cover different aspects of low mood. Items 2 and 3 appear alike, with Item 3 being broader.

### Synthesising Results and Choosing Items for a Shorter Scale

The results from these analyses suggest there are problematic items in the *paranoia* subscale, and several potentially redundant items in other subscales. A brief account of the reasoning for the removal of each item is provided as a table in Supplemental Table S2.

As a result of these analyses a shortened scale was produced containing the following items: Anthropophobia 1, 3, 4, Agoraphobia 1, 3, 4, Paranoia, 3,4,7, Lethargy item 3 (merged with depression subscale since lethargy is a symptom criterion of depression), Depression items 3, 4 and 5. Besides shortening each of the subscales, only one item from the *Lethargy* subscale was kept (question 3 which referred to the frequency of feeling of exhaustion) and was merged with the Depression subscale. After removing these items, 13 items remained, however it is important to note that the 3 best functioning paranoia items retained in the refined scale were still problematic in both the content analysis and the item analyses.

### Testing Performance of HRI Scale and Short HRI Scale

A CFA was conducted to examine the fit of several models of the HRI-24. The first, was a unifactorial model wherein all 24 items were loaded onto a single factor. The second model, HRI scales, was based on the five sub-scales of the HRI-24, and finally, the shortened HRI scale. The findings indicate a consistent enhancement in measurement quality by eliminating problematic items, with the shortened HRI model exhibiting the most favourable fit.

### Correlations Between HRI Scale, Internal Consistency Comparison and Gender Differences

The highly positive correlation between the HRI-24 scale and its shortened version (*r* = .98) suggests strong overall consistency. At a subscale level, the Pearson correlations were: Anthropophobia (Full vs. Short): *r* = .98, Agoraphobia (Full vs. Short): *r* = .98, Paranoia (Full vs. Short): *r* = .92 and Depression (Full vs. Short): *r* = .95. Additionally, given the removal of most lethargy items and the merging of the final item into the short HRI depression subscale, we assessed the relationship between the full Lethargy subscale and the short Depression subscale: Lethargy (Full) vs. Short Depression: *r* = .80.

The internal consistencies for the scales were similar on all scales except for paranoia. For the HRI-24, these were: Anthrophobia (α = .88), Agoraphobia (α = .88), Paranoia (α = .79), Lethargy (α = .84), Depression (α = .89) and overall α = .94. For the shortened version, the Cronbach alphas were: Anthrophobia α = .85, Agoraphobia α = .84, Paranoia α = .68 and Depression α = .87, and overall α = .91.

In the HRI-24, mean scores for men (*M* = 2.73, *SD* = 0.73) and women (*M* = 2.89, *SD* = 0.74) were similar. Although a statistically significant difference was found between the two groups, *t*(332.88) = 2.02, *p* = .044, the Bayes Factor (BF₁₀ = 0.85) indicated anecdotal evidence, suggesting the data are not sufficiently conclusive to support or refute a true group difference. Similarly, in the HRI-Short, women (*M* = 2.79, *SD* = 0.82) scored slightly higher than men (*M* = 2.62, *SD* = 0.79), with the difference approaching significance, *t*(329.75) = 1.97, *p* = .050. The Bayes Factor (BF₁₀ = 0.77) again provided only anecdotal evidence, implying weak support for any gender-based difference in short HRI scores.

### HRI-24 and HRI Short Associations with Related Measures

The correlations revealed significant positive associations between hikikomori risk and various neuroticism sub-scales, anxiety, depression, coping styles, shame and childhood factors. Negative correlations were found with approach coping and early life resources. No significant correlation was found between hikikomori risk and status ambition (Table 4).

**Table 4.** Comparing correlations between the HRI Short and HRI-24.

Variable	HRI short	HRI -24
Anxiety (neuroticism)	.547	.533
Anger (neuroticism)	.526	.542
Depression (neuroticism)	.725	.721
Self-consciousness (neuroticism)	.648	.640
Impulsiveness (neuroticism)	.272	.280
Vulnerability (neuroticism)	.666	.650
PhQ	.727	.739
GAD	.701	.705
Approach coping (BACQ)	−.457	−.468
Resignation and withdrawal (BACQ)	.735	.733
Diversion (BACQ)	.214	.227
Status anxiety	.629	.629
Status ambition	.066	.083
Unpredictable childhood	.384	.400
Early life resources	−.254	−.250
Internal shame	.703	.698
External shame	.705	.700

*Note.* The correlation between the HRI short and HRI 24 (*r* = .971).

In summary, the first study validated both the HRI-24 and the shortened HRI within a UK population, showing strong convergent and divergent correlations with relevant measures. Through item analysis, the HRI was reduced to 13 items, removing poorly functioning ones. Three subscales – *anthropophobia*, a*goraphobia* and *depression* – performed well, but issues were found with *paranoia* and *lethargy*. The *lethargy* subscale provided little new information beyond the most effective item, leading to its consolidation with *depression. Paranoia* contained many problematic items across analyses, and while three items remained in the shortened HRI, they showed issues in the Mokken analyses and failed to consider symptoms of paranoia beyond mistrust. The HRI short, retained a near-perfect correlation with the original scale (*r* = .98) and showed superior model fit in the CFA.

## Study 2

In Study 2, we examined the validity of the refined/shortened HRI measure by testing it against a measure of modern type depression (MTD). A positive correlation was anticipated, as MTD is considered a ‘gateway condition’ for hikikomori syndrome (Kato et al., 2019). MTD is characterized by depressive moods triggered by stress, avoidance in school and work, self-centeredness, low resilience and heightened vulnerability to trauma. Given that hikikomori is considered the most severe form of MTD, representing a new phenotypic presentation of youth depression (Orsolini et al., 2022), we predicted a statistically significant positive correlation between the HRI short and MTD, with a correlation in the range of *r* = .40 to .60 based on the shared psychosocial and emotional features between the two groups, and the mediating role MTD plays in the path from childhood maltreatment to hikikomori risk (Masuda et al., 2024).

### Participants

A total of 279 undergraduate psychology students participated in the study, recruited similarly to Study 1. Among them, 50 were males (*M* = 20.70, *SD* = 2.79) and 229 were females (*M* = 19.70, *SD* = 2.05). None of the participants had taken part in Study 1.

### Measures

#### Tarumi’s Modern Type Depression Trait Scale (TACS 22)

The TACS 22 (α = .80) is comprised of three subscales, Avoidance of Social Roles (α = .74), Complaint (α = .75) and Low Self-Esteem (α = .64) and demonstrated good test-retest reliability, convergent validity and 82.9% specificity for diagnostic accuracy for modern type depression (Kato et al., 2019).

#### HRI Short

The HRI Short is a 13 item is a shortened version of the HRI-24 following extensive IRT procedures and has four subscales to measure hikikomori risk. The Cronbach alphas for this sample were: *anthropophobia* (α = .79), *agoraphobia* (α = .74), *paranoia* (α = .53), *depression* (α = .80) and overall (α = .88)

#### Results

**Figure 1.**
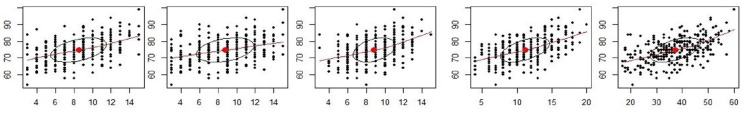
Scatter plots of HRI-short scales for anthropohobia, agoraphobia, paranoia, depression and total against TACS modern depression (vertical axis).

A moderate, positive correlation was found between the total scores of the shortened HRI (*M* = 37.2, *SD* = 9.68) and Modern Type Depression (*M* = 74.7 *SD* = 7.67) *r* = .50, *p* < .001. The correlations for HRI subscales with the total TACS22 ranged from *r* = .30 for the *agoraphobia* subscale, to *r* = .49 for the *depression* subscale (Figure 1). The HRI short with the depression subscale removed maintained a moderately positive association with Modern Type Depression (*r* = .45, *p* < .001).

## Discussion

This study had two objectives: first, to conduct an item analysis to shorten the HRI-24; and secondly, to observe the validity of the HRI-24 and the shortened HRI scale within an English-speaking UK population, since the measure was only validated in Italy and Japan (Colledani et al., 2023; Loscalzo et al., 2022). The IRT analysis found many problematic items in the *paranoia* subscale and identified weaker items within each of the other subscales, by identifying poorer scalability, lower correlations with withdrawal, lower factor loadings and discrimination values. The content analysis supported many of the IRT findings. After analyses, the HRI-24 was reduced to a 13-item scale, and only 4 sub-scales, merging the sole remaining lethargy item into the depression subscale.

A series of CFAs demonstrated that the HRI short had a superior fit than the HRI-24. The near perfect correlations between the HRI-24 and the shortened HRI support the validity of the shortened version. The HRI-24 (and HRI short) demonstrated strong convergent validity with significant positive correlations between hikikomori risk and all six of the neuroticism sub-scales, PHQ scores, GAD scores, the resignation and withdrawal coping, shame and unpredictable childhood. In contrast, there was a significant negative correlation between hikikomori risk and approach coping and between hikikomori risk and early life resources. The moderately positive correlation between the HRI short and the TACS 22, even when the depression subscale is removed, suggests further validity (Kato et al., 2019). These findings validate the HRI and HRI short for use in the UK. The correlation with MTD in the second study also indicates the measure reliably detects hikikomori risk, given the association between hikikomori and MTD (Orsolini et al., 2022).

Second, early life resources were negatively associated with hikikomori risk, suggesting they act as a protective factor (Varnum & Kwon, 2016). This could reflect relative poverty risk in wealthy countries, as a study in Japan found higher risk among poorer participants due to increased social security reliance (Imai et al., 2021). Lastly, similar to Loscalzo et al. (2022), no gender-based differences were found in hikikomori risk based on HRI-24 scores, which contrasts with prior studies (Kato et al., 2019; Pozza et al., 2019) that found it more common in men. These discrepancies may highlight either measure limitations or cultural differences in detecting hikikomori risk.

Malagón-Amor et al. (2018) highlighted gender disparities in hikikomori risk, noting more men overall, but more women in the anxiety-affective subgroup compared to those with psychotic or personality disorders. This finding is relevant given the strong correlations between the HRI-24 and negative affect measures like neuroticism, PHQ, GAD and shame. If a clinical definition includes subtypes, the HRI-24 primarily identifies the affective-anxious risk factors, potentially overlooking other risks. Given that hikikomori can occur without depression (Neoh et al., 2023), and that anxiety/depression severity isn’t always linked to social withdrawal (Benarous et al., 2022; Li & Wong, 2015), identifying risk factors beyond negative affect could improve the measure’s validity, including the possibility of egosyntonic presentations.

To improve the HRI-24 or HRI short, adding an item on self-efficacy could be valuable, as previous research links low self-efficacy to hikikomori risk (Uchida & Norasakkunkit, 2015). Item 2 of the lethargy subscale, which addresses ‘powerlessness’, showed strong factor loading, though it may not align with lethargy. The paranoia subscale may also need re-evaluation, as the remaining items focus on distrust, omitting other facets of paranoia, and the subscale had low Cronbach’s alpha. Despite these issues, the HRI-24 and HRI short demonstrated strong fit, validity, and reliability, particularly for the affective-anxious subtype of hikikomori.

### Limitations

While this research is novel and focuses on a new population, it does have limitations, including those associated with self-report measures. The participants were largely young adults, which differs from the broader age range (13–50 years) used in the original HRI-24 development and may limit the generalisability of our findings to older populations, however, given that hikikomori risk often emerges during adolescence and early adulthood, and previous studies have reported average onset in this age range, the sample remains relevant for examining early risk factors and symptom profiles associated with social withdrawal.

Additionally, participants were not screened for a hikikomori syndrome diagnosis. Future research could explore how correlates of HRI scores differ between individuals with low symptom levels and those presenting with clinical levels. Finally, although we chose not to include the NEET-Hikikomori Risk scale (NHR; Uchida & Norasakkunkit, 2015) – given its lack of validation in English and limited applicability outside Japan (Loscalzo et al., 2022) – future studies could benefit from adapting and validating the NHR for English-speaking populations. The NHR captures important sociocultural dimensions of hikikomori risk, such as the Freeter lifestyle, which refers to voluntary withdrawal from work or education. This behaviour is shaped by both individual motivation and broader factors like job market conditions and skills shortages, which differ significantly across countries. Including the NHR in future research would allow for a deeper exploration of how these sociocultural factors interact with the psychopathological elements captured by the HRI, such as social withdrawal, anxiety and depression, offering a more comprehensive understanding of hikikomori risk across different cultural contexts.

## Conclusion

Following a rigorous item analysis, we have developed a shortened 13-item version of the HRI-24, which we have shown is valid with a UK population. This is the first study to examine Hikikimori in the UK. Furthermore, we have demonstrated strong convergent validity between hikikomori risk and various measures of negative affect and discriminant validity with negative associations with protective factors, such as early life resources and approach coping. These findings underscore the need for refined measures to capture the multifaceted nature of hikikomori risk that could have clinical value when controlling for neuroticism. This short version could be useful in screening or epidemiological studies.

## Supplemental Material

sj-docx-1-isp-10.1177_00207640251348058 – Supplemental material for Hikikomori Risk in the UK Supplemental material, sj-docx-1-isp-10.1177_00207640251348058 for Hikikomori Risk in the UK by Gregory Gorman, Alison Bacon, Jon May and Stephen Minton in International Journal of Social Psychiatry

sj-docx-2-isp-10.1177_00207640251348058 – Supplemental material for Hikikomori Risk in the UK Supplemental material, sj-docx-2-isp-10.1177_00207640251348058 for Hikikomori Risk in the UK by Gregory Gorman, Alison Bacon, Jon May and Stephen Minton in International Journal of Social Psychiatry
