# Supplementary material for: Hikikomori Risk in the UK
Source: Int J Soc Psychiatry. 2025 Jul 5;71(8):1621–32. doi: 10.1177/00207640251348058 (PMC12634888; doi:10.1177/00207640251348058)
Supplement: sj-docx-1-isp-10.1177_00207640251348058 – Supplemental material for Hikikomori Risk in the UK [file sj-docx-1-isp-10.1177_00207640251348058.docx]

**Item Information Functions**

The Item Information Functions (IIFs) depict how much information each item provides at varying levels of the latent trait (θ). The x-axis represents the level of the trait (ranging from very low to very high), and the y-axis indicates the amount of information contributed by each item. Peaks reflect the θ levels where items are most informative.


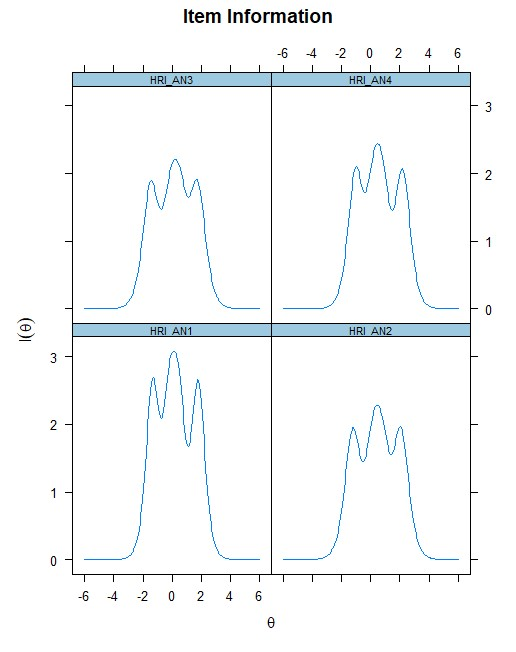


**Fig. S1.** Item Information Function of Anthropophobia


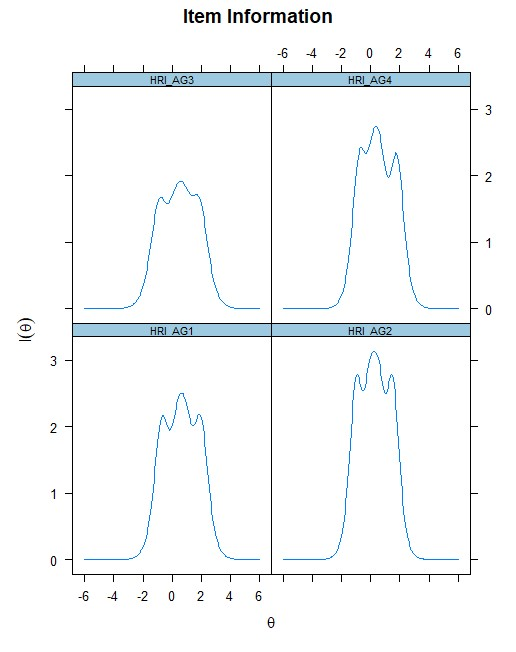


**Fig.S2.** Item Information Function of Agoraphobia


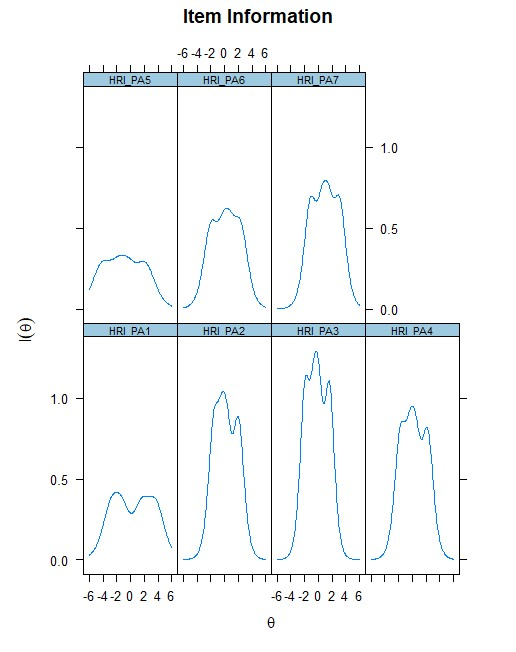


**Fig.S3.** Item Information Functions of Paranoia.

 
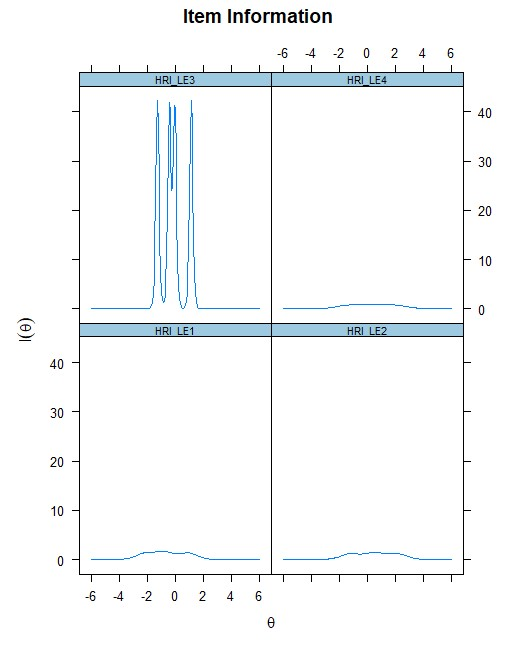


**Fig.S4.** Item Information Function of Lethargy


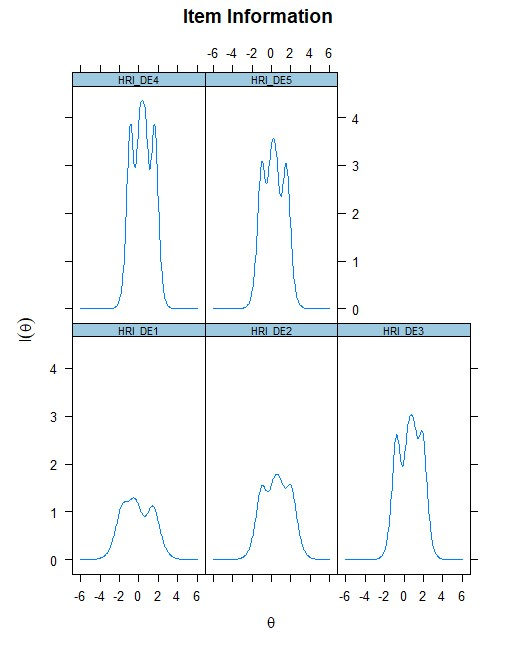

**Fig.S5**. Item Information Function of Depression
