# Supplementary material for: Hikikomori Risk in the UK
Source: Int J Soc Psychiatry. 2025 Jul 5;71(8):1621–32. doi: 10.1177/00207640251348058 (PMC12634888; doi:10.1177/00207640251348058)
Supplement: sj-docx-2-isp-10.1177_00207640251348058 – Supplemental material for Hikikomori Risk in the UK [file sj-docx-2-isp-10.1177_00207640251348058.docx]

**Table S1**

*Content Analysis of each item and definitions and symptoms of each condition*

| **Sub-scales** | **Definition and symptoms** | **Item** | **One word content** |
| --- | --- | --- | --- |
| *Anthropophobia* |  |  |  |
|  | Fear of people, being judged, not meeting standards, feeling that you will offend others and be watched. Negative comparison to others | 1. I feel severely anxious when I meet people outside | Anxiety. People outside |
|  |  | 2. I am uncomfortable when I am in touch with others | Discomfort. Phrasing issue. |
|  |  | 3. I have great difficulty in participating in social activities | Difficulty w/ others |
|  |  | 4. I am afraid of social relationships | Fear of people |
| *Agoraphobia* |  |  |  |
|  | Fear of open spaces where escape may be difficult, or help is unavailable. Avoidance behaviour is common. Might be related to fear of fear of crimes/accidents. | 5. I feel very anxious when I am in a closed space (e.g., shops, cinema, theater, bank, etc.) | Anxiety. Public space |
|  |  | 6. I am afraid of being in crowded places | Fear crowded space |
|  |  | 7. I avoid going to public places when I go out alone | Avoidance. Public spaces |
|  |  | 8. When I am in crowded places, I feel some strange sensations (accelerated heartbeat, excessive sweating, tremors, choking sensations, etc.) | Physical sensations |
| *Paranoia* |  |  |  |
|  | Feeling people dislike you, will harm or criticize you. Distrust of others. Persecution. Easily offended and difficulty getting on with others. | 9. I am diffident and suspicious of others | Distrust. Double barrelled question. Phrasing issue. |
|  |  | 10. Confiding with others is dangerous because people might then reveal my secrets | Fear and distrust. Secrets. Double barrelled question. |
|  |  | 11. I am afraid of being deceived by others | Fear and distrust |
|  |  | 12. I prefer doing things on my own because I do not trust people | Autonomy and distrust |
|  |  | 13. People are willing to do anything to achieve their goals | Cynicism, others are ambitious |
|  |  | 14. I only trust myself | Autonomy and distrust |
|  |  | 15. If others do me a favor, I do not believe in their good intention | Distrust |
| *Lethargy* |  |  |  |
|  | Impact of tiredness of mood, physical symptoms maybe like headaches or mood. | 16. I feel tired and fatigued | Exhaustion |
|  |  | 17. I manage to do my activities with difficulty because I feel powerless | Low self-efficacy or agency |
|  |  | 18. I often feel weak and lacking in energy | Weakness |
|  |  | 19. I sleep many hours a day because I often feel weak | Excessive sleep. Weakness. Two constructs |
| *Depression* |  |  |  |
|  | Continuous low mood, helpless, hopeless, low self-esteem, shame/guilt no motivation, irritable, no enjoyment, suicidal thoughts. Lack of energy/lethargy. Unexplained pains. | 20. There are moments in which nothing seems important | Apathy. Indifference/values |
|  |  | 21. Nothing is able to really thrill me | Apathy. Lack excitement/feeling |
|  |  | 22. I rarely feel positive emotions | Apathy/ feelings |
|  |  | 23. I feel dispirited and hopeless for the future | Hopeless |
|  |  | 24. I feel a sense of inner emptiness | Emptiness |

**Table S2**

*Rationale for removing items.*

| **Subscale** | **Items** | **Reasons for removal** |
| --- | --- | --- |
| *Anthropophobia* | 2 | Question 2 shows worst discrimination, IFF function, and scalability. Unclear phrasing with similarity in construct around socializing difficulties as in Q3. |
|  |  |  |
|  |  |  |
| *Agoraphobia* | 2 | Questions 1 and 2 address anxiety regarding public spaces. Although item 2 performed better in the IFF on a subscale level, item 1 had better fit with the overall scale and BACQ withdrawal. |
|  |  |  |
|  |  |  |
| *Paranoia* | 1,2, 5,6 | Q1 employs inaccessible language and performs poorly across analyses. Q2 is a double-barrel question with low discrimination and scalability. Q5 aligns more with cynicism than paranoia. Q4 and Q6 both concern autonomy and distrust, but Q4 shows better scalability, factor loading, and discrimination. |
|  |  |  |
|  |  |  |
| *Lethargy* | 1,2,4 | Content analysis and IFF indicate that only Item 3 effectively explains information within this sub-scale, specifically addressing lethargy. Its information curve signifies its unique ability to discriminate information compared to the other items, which cover a similar construct but less effectively. Item 2 is about powerlessness rather than lethargy. |
|  |  |  |
|  |  |  |
| *Depression* | 1, 2 | Items 1 and 2 show similar difficulty levels in IFF but lower discrimination than other items. Content analysis indicates some redundancy in the aspects of depression measured by these items. |

**Table S3**

*Comparing correlations between the HRI short and HRI-24*

|  | **HRI Short** | **HRI -24** |
| --- | --- | --- |
| Anxiety (Neuroticism) | 0.547 | 0.533 |
| Anger (Neuroticism) | 0.526 | 0.542 |
| Depression (Neuroticism) | 0.725 | 0.721 |
| Self consciouness (Neuroticism) | 0.648 | 0.640 |
| Impulsiveness (Neuroticism) | 0.272 | 0.280 |
| Vulnerability(Neuroticism) | 0.666 | 0.650 |
| PhQ | 0.727 | 0.739 |
| GAD | 0.701 | 0.705 |
| Approach coping (BACQ) | -0.457 | -0.468 |
| Resignation and withdrawal (BACQ) | 0.735 | 0.733 |
| Diversion (BACQ) | 0.214 | 0.227 |
| Status anxiety | 0.629 | 0.629 |
| Status ambition | 0.066 | 0.083 |
| Unpredictabile childhood | 0.384 | 0.400 |
| Early Life Resources | -0.254 | -0.250 |
| Internal Shame | 0.703 | 0.698 |
| External Shame | 0.705 | 0.700 |

*Note.* The correlation between the HRI short and HRI 24 (r = .971)
